# A Unique Combined Ganglioneuroma Schwannoma Tumor Mimicking Adrenal Malignancy

**DOI:** 10.7759/cureus.5500

**Published:** 2019-08-27

**Authors:** Katherine R Porter, Seema Shroff, Tien-Anh Tran, Vladimir Neychev

**Affiliations:** 1 Miscellaneous, University of Central Florida College of Medicine, Orlando, USA; 2 Pathology, AdventHealth, Orlando, USA; 3 Surgery, University of Central Florida College of Medicine, Orlando, USA

**Keywords:** ganglioneuroma, schwannoma, adrenal incidentaloma

## Abstract

A 28-year-old woman with a past medical history significant for cervical cancer was diagnosed with a 2.5 cm adrenal tumor but was lost to follow-up. Two years later, she presented to the emergency room with worsening right upper abdominal and flank pain. The computed tomography (CT) and magnetic resonance imaging (MRI) of the abdomen and pelvis revealed that the right adrenal mass had nearly doubled in size (4.3 cm), was heterogeneous with calcifications, central necrosis and actively uptaking the intravenous (IV) contrast with a delayed washout. The biochemical workup was negative for hyperaldosteronism, hypercortisolism, and pheochromocytoma. She reported an unintentional body weight loss of 40 pounds. Adrenocortical carcinoma or a metastatic malignancy was high on the differential diagnoses list. She underwent a successful laparoscopic adrenalectomy, and final pathology revealed a benign extra-adrenal combined ganglioneurofibroma and schwannoma. These rare benign malignancies alone or in combination may closely mimic the clinical and imaging characteristics of adrenal malignancy and pose a diagnostic and therapeutic dilemma to surgeons as well as cause a significant distress to patients and their families. Thus, it is important to thoroughly document and report these cases in order to increase awareness and improve our understanding of the biology, natural history and management of these extremely rare tumors.

## Introduction

The overall prevalence of incidentally found adrenal lesions is roughly 4.4%; however, this prevalence varies with the patient’s age, and incidentalomas occur in less than 1% of patients younger than 30 years old [1]. The vast majority of adrenal incidentalomas are asymptomatic, which is why they most often present in the context of imaging for an unrelated illness. It has been shown that 80% of incidentalomas are benign adenomas, 7% are metastases, and 4% are ganglioneuromas [2]. The two major predictors of malignancy are the tumor size and its appearance on imaging [3]. The mass size cutoff of 4 cm has a 93% sensitivity for primary adrenocortical cancer [4]. Adrenal metastases typically demonstrate irregular shape and heterogeneous nature with necrosis and calcifications, whereas benign adenomas typically appear round and homogenous with smooth contour and sharp margination [5, 6].

Here we present a case of benign combined ganglioneurofibroma and schwannoma, with clinical signs, pre-operative imaging, and intra-operative observations highly suggestive of a malignant adrenal mass.

## Case presentation

A 28-year-old woman with a history of cervical cancer was diagnosed with a 2.5-cm right adrenal mass; however, the patient was lost to follow up. Two years later, she presented to the emergency department complaining of knee pain, and worsening back and bilateral flank pain. She reported experiencing pain over the past two years, but it had worsened and become constant over the past two days. She also reported episodes of headaches, palpitations, hand tremors, and sweating, which had been increasing in frequency and severity over the past 2-3 years. She admitted to regular tobacco and marijuana use and a significant unintentional weight loss of about 40 pounds. She denied alcohol use. She had a past medical history significant for anemia and stage I cervical cancer, and she denied any history of hypertension. Her family history was noncontributory.

At initial evaluation, her body temperature was 98.5^ᵒ^F, pulse rate of 117 beat per minute, and hypertensive, with a blood pressure of 140/99. The abdominal exam revealed right-sided tenderness on palpation, and the musculoskeletal exam revealed bilateral lumbar tenderness and right lower leg pain with movement. There were normal bowel sounds on auscultation; no organomegaly, abdominal wall defects or abdominal distension. The remainder of the physical exam was unremarkable. The laboratory evaluation was significant for decreased red blood cell count of 3.64 x 10^6^/µL, mild anemia of 11.2 g/dL, mild hypocalcemia of 8.4 mg/dL, hypoproteinemia of 6.1 g/dL, increased blood glucose of 117 mg/dL, and urine nitrite and bacteria.

**Figure 1:**
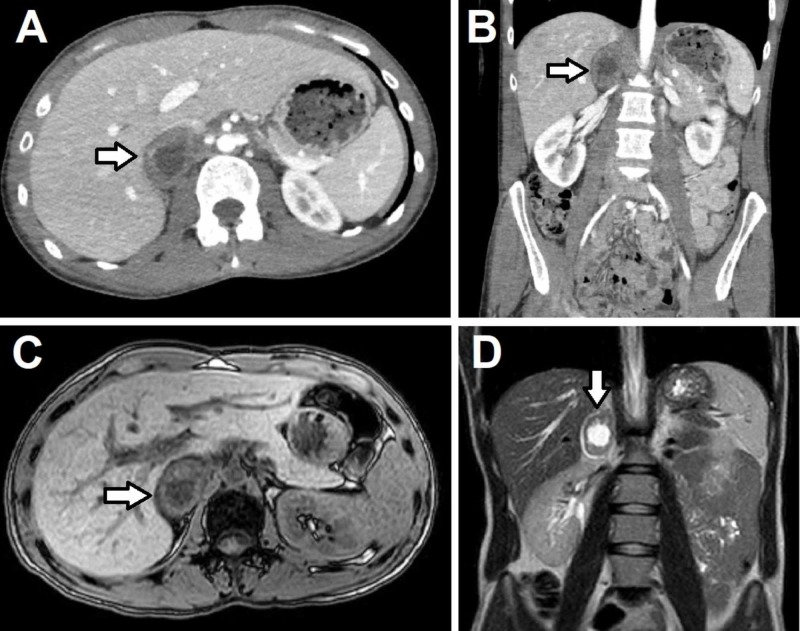
Computed tomography (CT) and magnetic resonance imaging (MRI) of the abdomen and pelvis (A) Axial and (B) coronal view of the CT scan of the abdomen and pelvis. (C) Axial view of the In and Out phase of the MRI of the abdomen and pelvis and (D) coronal view of the T2 phase of the MRI of the abdomen and pelvis. The arrows are pointing at a 3.7 cm x 2.9 cm x 4.3 cm heterogeneous mass in the right adrenal gland with central necrosis, irregular borders, calcifications and significant compression of the inferior vena cava.

Computed tomography (CT) of the abdomen and pelvis revealed a 3.7 x 2.9 x 4.3 cm thick-walled and centrally necrotic mass on the right adrenal gland, which appeared to be exerting mass effect on the inferior vena cava (IVC) (Figure 1A, 1B). Magnetic resonance imaging (MRI) of the abdomen was ordered to better define the relationship of the tumor to the IVC and renal hilum, and it revealed that the mass had irregular walls and was pushing on the retrohepatic IVC with poorly defined planes between the tumor and the IVC wall (Figure 1C, 1D). Due to border irregularities and central necrosis, as well as the patient’s history of cervical cancer, the tumor was highly suspicious for metastatic disease or an adrenocortical carcinoma (ACC). Pheochromocytoma was also considered in the differential due to the patients’ complaints of headaches, palpitations, tremors and sweating. Biochemical testing of the tumor for hypercortisolism, hyperaldosteronism, and catecholamine oversecretion (pheochromocytoma) revealed no hormonal oversecretion. The possible diagnoses were discussed in detail with the patient, and surgical removal of the tumor was decided as the ultimate goal.

The risks and benefits of laparoscopic versus open adrenalectomy were discussed in detail, and written informed consent was obtained from the patient. A successful laparoscopic right adrenalectomy was performed following the surgical oncological principles. Intraoperatively, ACC or metastasis remained the main differential diagnoses.

**Figure 2:**
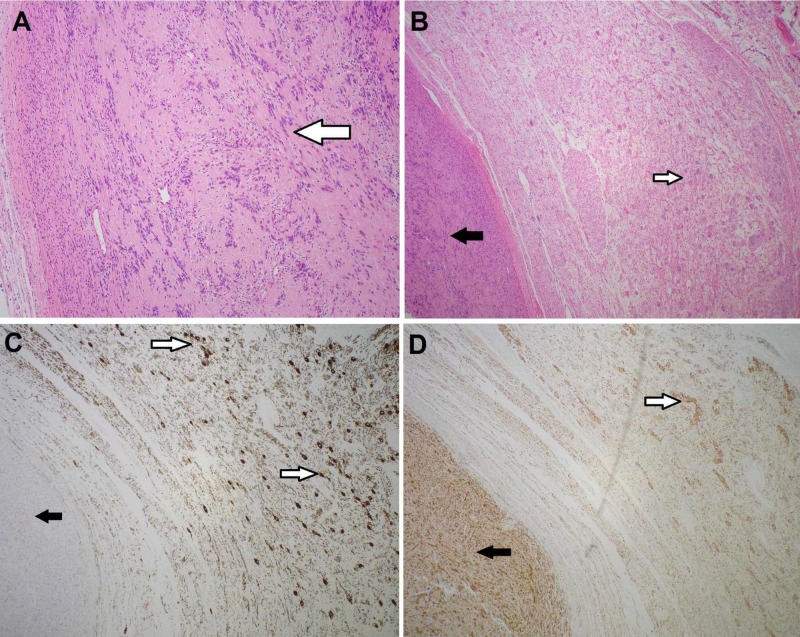
Histological staining of extra-adrenal mass consistent with combined ganglioneuroma schwannoma tumor (A) Central component of the tumor (schwannoma) composed of spindle cells with wavy nuclei set in a collagenous background (white arrow). (B) Schwannoma, representing the central component of the tumor (black arrow); Ganglioneuroma, representing the peripheral part of the tumor (white arrow). (C) Neurofilament (NeuN) immunostaining positive in ganglion cells (white arrows), negative in the schwannoma (black arrow). (D) S100 immunostaining - positive in both components of the tumor (black arrow - schwannoma; white arrow - ganglion cells).

Surgical pathology, however, revealed an extra-adrenal combined ganglioneurofibroma and schwannoma with no malignant features (no atypia, no mitosis). The specimen was an intact adrenal gland, with a well circumscribed, encapsulated gray/tan mass measuring 27.6 g intimately attached to the adrenal cortex. The remaining adrenal gland, including the medulla, is intact and uninvolved. The tumor demonstrated a central nodule composed of spindle cells with wavy nuclei set in a collagenous background (Figure 2A). The central nodule was surrounded by a peripheral component composed of proliferation of spindle cells set in loose myxoid stroma with multiple ganglion cells (Figure 2B). Immunohistochemical stain showed that only ganglion cells in the peripheral part of the tumor were positive for neurofilament (NeuN) immunostaining (Figure 2C), while both the central nodule and the periphery were positive for S100 immunostaining (Figure 2D). These histological and immunohistochemical features were consistent with a combined schwannoma and ganglioneuroma, with clear division between the two cell types. The remaining adrenal gland was unremarkable.

The patient tolerated and recovered from surgery well and was discharged to home on day 2 after surgery.

## Discussion

It has been shown that isolated ganglioneuromas or schwannomas are rare and may be seen in approximately 1.4% of adrenal incidentalomas, respectively [7]. A combined ganglioneuroma and schwannoma is extremely rare, and to our knowledge this is the first case found in the adrenal gland reported in the literature.

The underlying pathophysiology of this specific form of adrenal mass has not been fully elucidated, especially in patients without neurofibromatosis type 2 (NF2). In patients with NF2, there is a mutation in the NF2 gene, located on chromosome 22, which normally produces a tumor-suppressor protein merlin [8]. Patient’s family history was not contributory, and she did not have the clinical stigmata of NF2 including a history of previous schwannomas or juvenile cataracts. Ganglioneuromas typically occur in older children, and they are considered the benign counterpart of neuroblastoma. They typically present with a thoracic mass, rarely occurring in the adrenal gland [9]. In general, adrenal tumors occur most commonly in adults 60-80 years old [1].

Although benign adrenal masses account for the vast majority of adrenal incidentalomas, the prevalence is extremely low in patients less than 30 years old [1]. Additionally, this patient presented with a history of cervical cancer and a tumor which had nearly doubled in size over the previous two years and was centrally necrotic with irregular borders. On pre-operative imaging, the tumor’s relationship with the IVC was poorly defined. It was unclear if the tumor was invading the vessel, or simply pushing against it. Finally, the patient exhibited clinical signs of a hormone secreting tumor - headaches, tremors, palpitations, sweating, and markedly elevated blood pressure and blood glucose levels. The size of the tumor is often used as a predictor of malignancy, and in this case, the tumor's size - 4.3 cm at its widest diameter - would have supported its malignancy.

In this case, the diagnosis of a benign mass was the best possible outcome for the patient, that does not require follow-up or other treatments after surgery. However, it is important to note that cases can occur exactly opposite to this one - where all clinical signs and studies point to a benign mass, but malignancy is discovered on histological examination. Thus, this case highlights the importance of maintaining an open and dynamic differential diagnosis, which can ultimately lead to the best care and outcome for the patient.

## Conclusions

This case will contribute to increasing awareness and better understanding of the complexities that surround neuroendocrine tumors. Distinguishing between the various types of adrenal masses remains difficult and rather ambiguous pre-operatively, thus post-operative pathology is critical to determining the patient’s subsequent treatment plan, should they require one. Treatment decisions should be made through a collaborative team effort with surgeons, pathologists, radiologists, and oncologists to ensure the most favorable outcome for the patient.
